# The role of dendritic cells in MASH: friends or foes?

**DOI:** 10.3389/fimmu.2024.1379225

**Published:** 2024-04-08

**Authors:** Antonio T. Pinto, Veronika Lukacs-Kornek

**Affiliations:** Institute of Molecular Medicine and Experimental Immunology, University Hospital Bonn of the Rheinische Friedrich-Wilhelms-University, Bonn, Germany

**Keywords:** dendritic cells, liver, MASH, inflammation, metabolic dysfunction associated fatty liver disease

## Abstract

Dendritic cells (DCs) are major antigen-presenting cells that connect innate and adaptive immunity. Hepatic DCs are less activated and contribute to maintain the tolerogenic environment of the liver under steady state. Several studies indicated DCs in metabolic dysfunction-associated steatohepatitis (MASH), representing a substantial burden on healthcare systems due to its association with liver-related morbidity and mortality. Studies highlighted the potential disease-promoting role of liver DCs in the development of MASH while other experimental systems suggested their protective role. This review discusses this controversy and the current understanding of how DCs affect the pathogenesis of MASH.

## Introduction

Metabolic dysfunction-associated steatohepatitis (MASH) has recently become a significant health issue, presenting as a progressive and potentially severe form of metabolic dysfunction-associated fatty liver disease (MAFLD) (1, 2). This intricate condition is characterized by hepatic inflammation and liver cell damage and association with liver-related morbidity and mortality (3). During the progression of MAFLD caused by liver steatosis, patients are at risk for multiple complications, including hypertension, atherosclerosis, and other diseases (4). In addition, MASH has the potential to progress to a more advanced form of liver disease, such as cirrhosis and hepatocellular carcinoma (HCC) (5).

The pathogenesis of MASH is a multifaceted process involving hepatocyte triglyceride accumulation followed by additional triggering factors, including oxidative stress, lipotoxicity, mitochondrial dysfunction, and pro-inflammatory cytokine release, that contribute to the worsening of the condition (6). Hepatic lobular inflammation is a defining characteristic of the transition from simple steatosis to steatohepatitis. Presently, it is recognized as the primary factor driving the progression of MAFLD to MASH (7), as well as the development of cirrhosis and its specific contribution to extrahepatic complications associated with the disease (5, 8). Prolonged inflammation aggravates tissue damage and may lead to an abnormal response in tissue healing, thereby contributing to the emergence of MASH and liver fibrosis. In this context, as a response to metabolic-related stress, both the innate and adaptive immune system become activated. Understanding the interaction between immune and parenchymal cells is the key challenge in deciphering the pathogenesis of the disease (6, 9).

## Liver as a multipurpose organ with complex immune and dendritic cell network

The liver is well known for its complex metabolic function and its role in the maintenance of homeostasis. Structurally, the liver is composed of repeating functional units that are defined by the vascular supply structures. These structures form a network of sinusoidal vessels that provide blood to the metabolic cellular units of the liver: hepatocytes and cholangiocytes (10). The hepatocytes are separated from the bloodstream by non-parenchymal cells, including a layer of fenestrated liver sinusoidal endothelial cells (LSECs) that lack a basement membrane. Hepatic stellate cells (HSCs) are also present in the small space of Dissé between LSECs and hepatocytes (10). Besides these parenchymal cells, the liver is home for multiple subsets of immune cells, e.g., macrophages, dendritic cells (DCs), classical T and B lymphocytes, and innate lymphoid cells (10–12). Recent years of scRNAseq and multiparameter flow cytometry analyses revealed diverse immune cell types present in the liver, but more importantly, it highlighted the manifold subset variety that could be identified (13–15). Based on extensive single-cell analyses, novel tools have also been developed such as the Liver Cell Atlas that are available for scientists to survey their molecule of interest (13, 14). It is becoming more evident that the presence of such complex immune network forms an interconnected functional unit with the metabolic and parenchymal liver tissue under both physiological and pathological conditions.

Among DCs, we distinguish different DC subtypes based on their origin and phenotypic criteria (16). Conventional DCs (cDCs) develop from hematopoietic stem cells in the bone marrow via intermediate stages as common DC progenitor (CDP) and pre-cDCs. This process is dependent on Flt3L–Flt3 interaction (17). Pre-DCs leave the bone marrow and colonize lymphoid and non-lymphoid organs and become conventional DC type 1 (cDC1) and conventional DC type 2 (cDC2) cells. The two subsets depend on different transcription factors for their development. cDC1s need, e.g., *Irf8, Batf3, Id2*, and *Nfil3* while cDC2s need *Irf4* and *Irf2* (18). They also differ in their surface markers used to identify and distinguish these cells (**Table 1**). cDC1s in murine lymphoid organs express CD8α, but in non-lymphoid organs, they are rather positive for CD103 or CD24. Murine cDC2s are more abundant than cDC1s but represent a very heterogeneous cell population (16, 18). Using scRNAseq analyses, studies identified DCs expressing BDCA-3 (blood dendritic cell antigens 3) and XCR1 (X-C motif chemokine receptor 1) as cDC1s in humans (19–21). Human cDC2s lack the above canonical markers and exhibit heterogeneous populations with different gene expression profiles across tissues (19–23). High-resolution scRNAseq identified further putative cell clusters that may correspond to additional human cDC subsets (16, 22, 24). A recent study demonstrated DC3 population derived from Ly6C^+^ monocyte-dendritic cell progenitors representing a novel DC subtype (24). Further studies are necessary to clarify these clusters and how do they relate to other DC subsets.

**Table 1:** The summary of surface markers of DC subsets.

	Mouse	Human
**cDC1**	CD8α^+^ (lymphoid organs) CD103^+^, CD24^+^ (non-lymphoid organs) CD11c^+^ MHCII^+^ XCR1^+^ CLEC9A^+^ CD11b^-^ SIRPα^-^	HLA-DR^+^ CD141/BDCA-3^+^ CD123^-^ CD11c^+^ CD14^-^ XCR1^+^ CLEC9A^+^ SIRPα^-^ CD1a-
**cDC2**	CD11c^+^ MHCII^+^ CD11b^+^ SIRPα^+^ XCR1- CLEC9A-	HLA-DR^+^ CD1c/BDCA-1^+^ CD123^-^ CD11c^+^ CD14^-/+^ XCR1^-^ CLEC9A^-^ SIRPα^+^ CLEC10A^+^ CD16^-^ CD32^hi^ CD5^+/-^ CD14^-^ CD272^hi^ CD36^low^
**DC3**	SIRPα^+^ Lyz2^+^ CD16/32^+^	CD163^+^ CD36^hi^ CD14^+/-^ CD16^-^
**pDC (ILCs)?**	BST2^+^ B220^+^ SiglecH^+^ CD11^low^ CD11b^-^	HLA-DR^+^ CD303/BDCA-2^+^ CD123^+^ CD11c^+^ CD45RB^+^ CD304/Neuropilin^+^
**Mo-DC (inflammatory DCs)**	CD11c^+^ MHCII^+^ CD11b^+^ SIRPα^+^ Ly6C^+^ CD206^+^ FcεR1^+^ CD64^+^ F4/80^+^	HLA-DR^+^ CD1c/BDCA-1^+^ CD1a^+^ CD14^+^ CD11c^+^ CD11b^+^ FcεR1^+^ SIRPα^+^

ILCs, innate lymphoid cells.

Resting DCs receive environmental signals (e.g., type I IFNs) that maintain their homeostasis and survival (25, 26). cDCs are able to sense the environment via innate immune receptors such as pathogen-associated molecular patterns (PAMPs) and danger-associated molecular patterns (DAMPs) or respond to various cytokines (e.g., Il1β) (27, 28). Response to such danger signals is key in the initiation of immune responses (29). DCs upon danger signal become activated, a process referring to the ability of DCs to become capable of delivering information to other immune and/or non-immune cells (16). DCs are sampling the environment and able to migrate to draining lymph nodes to present antigens themselves on MHC-I or on MHC-II molecules or hand over antigens to resident cDCs for presentation (30, 31). Based on their processing machinery, cDC1s were considered to be superior in CD8 T-cell priming and specialized in cross-presentation while cDC2s favored CD4 T-cell priming (32). Several studies indicated that these differences are not due to intrinsic ability but rather differences in location and antigen access and both subsets of cDCs are able to activate both types of T cells (33, 34).

In addition to their role as initiators of adaptive immunity, cDCs are involved in immune tolerance both in the thymus and at the periphery (35). Notably, tolerogenic DC activation was considered as a semi-activated state compared to the fully activated DCs evolved upon PAMP engagement. Growing lines of evidence suggest that tolerogenic DC activation represents not a different level of quantitative activation but an entirely complex gene expression profile enabling cDCs to use a different set of effector capacities to interact with the environment (36, 37).

One of the most distinguished ability of cDCs is the activation of naïve and memory T cells and thereby the initiation of adaptive immune responses. They also can directly present antigens to B cells, leading to humoral response and antibody production. Additionally, DCs interact with NK and NKT cells and connect innate and adaptive immunity (16, 38). Besides immune cells, DCs connect the immune and non-immune compartment to orchestrate the organ-specific responses. cDCs communicate with fibroblastic reticular cells and help adjust the stromal compartment during priming (39). They also produce VEGF or IL31 for endothelial and neuronal crosstalk (40, 41). Thus, cDCs represent a central component in the interconnective immune and non-immune network during immune responses.

Plasmacytoid DCs (pDCs) were long considered as a third subtype of DCs originating from a lymphoid progenitor distinct from the one that gives rise to cDCs (42). pDCs are capable of producing a large amount of type I IFNs upon infection, and their role in T-cell activation and tolerance is still debated. Recently, they have been suggested for re-classification as innate lymphocytes (43).

Monocyte-derived DCs (Mo-DCs) are positive for CD11c and MHCII and they were believed to serve as antigen-presenting cells especially during inflammation (44, 45). Mo-DCs express genes associated with inflammation, but their role in T-cell priming is questionable. A recent study demonstrated that highly purified Mo-DCs are unable to perform these tasks (46).

While DC physiology is well studied in lymphoid organs, it is less understood in the liver microenvironment.

## Hepatic dendritic cells: functional dichotomy under steady state

Under steady state, hepatic DCs are relatively sparse in the liver (make up approximately 1% of the non-parenchymal cells) and mostly located in the periportal area. Here, they form an interconnected network where cDC1s mostly localized along the lymphatic vessels, while cDC2s surround the biliary tree (10, 47), suggesting functional division among subsets. DCs closely interact with the microenvironment in the portal area, respond to extracellular matrix (ECM) signals with maturation, and migrate to draining hepatic lymph node (48, 49). In this context, DCs as sentinels of the immune system play a central role in the initiation, modulation, and resolution of hepatic inflammation (10). Being strategically positioned within the liver allows liver DCs to survey and respond to changes in the hepatic microenvironment, making them indispensable contributors to the orchestration of immune responses in the liver. The liver, as most non-lymphoid organs, also possesses DC precursors with the capacity to differentiate towards all three DC subtypes (10, 50). Additionally, it has been reported that CD11c^+^ CX3CR1^+^ DCs, representing an additional DC subset in the liver, are located at the subcapsular area and replaced by monocytes from the circulation (51). What is the exact role of these cells and how they relate to other conventional DC subsets in liver homeostasis and pathology remain to be elucidated.

Liver cDCs under steady state have a lower capacity to endocytose antigen and are less efficient in allogenic T-cell activation than lymphoid DCs. They express immunosuppressive cytokines such as interleukin-10 (IL-10) and transforming growth factor-beta (TGF-β). To complement the liver tolerogenic environment, they respond less to LPS (and other TLR) stimuli: a phenomenon referred to as endotoxin tolerance (10, 50, 52, 53). pDCs, on the other hand, primarily promote Treg responses in the liver and are crucial in the tolerogenic response to oral antigens (10, 52, 53). Thus, overall, these specialized DC subsets in the liver act as APCs and modulate immune responses by promoting the generation/function of Tregs, by inhibiting pro-inflammatory T-cell responses, and by supporting the maintenance of immune tolerance under steady state via their unique cytokine profile.

## MASH and the adaptive arm of the immune response

A large body of evidence suggests that the activation of adaptive immune components contributes significantly to the progression and modulation of inflammation in MASH, influencing the disease pathogenesis and outcomes. It is well acknowledged that patients with MASH exhibit notably elevated levels of hepatic cytotoxic CD8^+^ T cells in comparison to healthy individuals (54–56). This is accompanied by the elevated presence of Th17 and reduced numbers of Tregs in the liver, promoting a pro-inflammatory milieu (57, 58). Rag1KO animals that lack mature B cells, T cells, and NKT cells are unable to mount adaptive T-cell responses and have greatly attenuated MASH (59). Mice deficient in β2m, lacking CD8^+^ T cells, exhibit protection against both steatosis and MASH upon choline-deficient high-fat diet (CDHFD) challenge. This protection relates to a decrease in the production of LIGHT by CD8^+^ T cells and NKT cells and the consequently reduced hepatocyte damage (59). Accordingly, the increase in CD8^+^ T cells and the reduction in the CD4 T-cell compartment contribute not only to the progression of MASH but also to the transition of MASH to HCC (59, 60). In recent years, it became clear as well that CXCR6^+^granzyme-B^+^PD1^+^ effector/memory phenotype CD8 T cells accumulate in the liver during MASH (61). These cells became metabolically activated by acetate and extracellular ATP in the microenvironment. This leads to their autoaggressive behavior that resulted in hepatocyte death that was MHC-I independent and mediated via purinergic receptor signaling (P2X7) (61). Together, these data suggest that primed T cells homing to the liver are responsible for the liver damage and MASH. Metabolic activation of T cells is further promoted by B cells independent of TCR signaling (62). B cells become activated within the lamina propria independent of microbiota and home to the liver where they affect CD8 T cells and further promote damage and fibrosis via their IgA secretion (62). This phenomenon is accompanied by the increased presence of autoantibodies targeting oxidative stress-derived epitopes in approximately 40% of adults and in 60% of children with MASH (63). These lines of evidence suggest that MASH is highly mediated and dependent on the adaptive immune response. The question is how DCs, which are the major APCs linking innate and adaptive immunity, fit in this picture.

## The role of DCs in MASH: the cDC1 controversy

The role of DCs is highly controversial and still remain not well understood in MASH (**Table 2**). Increased DC (cDC1, cDC2, and pDCs) infiltration is a hallmark of human MASH (56, 68) and has been demonstrated in multiple animal models (64–66). The involvement of cDCs in MASH has been established in the study conducted by Henning et al., in which DCs exhibited a more activated phenotype and the ablation of DCs using BM chimeric CD11c.DTR mice resulted in a larger influx of inflammatory cells to the liver, increased production of various cytokines associated with hepatic injury, and accelerated hepatic fibrosis (64). These findings suggested that cDCs may have a protective role in the context of disease. This was further highlighted using animal model deficient in the transcription factor Batf3 (basic leucine zipper ATF-like transcription factor 3) that lacks CD103^+^ cDC1s. Batf3 KO animals demonstrated a switch towards MASH from steatosis under high sucrose diet (HSD) including an increased presence of inflammatory infiltrates, steatosis, and elevated hepatocellular damage compared to WT animals (65). CD11c^+^CD11b^+^ myeloid cells showed increased frequency in Batf3 KO HSD fed animals, and the production of cytokines including IL-1ra, CCL2, CXCL1, CCL5, and TNF was also elevated (65). In addition, upon adoptive transfer of bone marrow-derived CD103^+^ cDC1s, a notable reduction was observed in the influx of pro-inflammatory monocytes and TNF production by CD11c^+^ cells (65). Thus, both the above studies identified DCs that balance the pro- and anti-inflammatory environment and regulate the presence of the inflammatory infiltrates. Both studies, however, used systems that were not exclusively specific for DCs. CD11c is a marker that could also be expressed by, e.g., NK cells, inflammatory monocytes, and neutrophils, while Batf3 could be present in regulatory T cells, gamma/delta T cells, and type 9 helper T cells (16, 69, 70). Therefore, the effect of Batf3 ablation or depletion of CD11c^+^ cells could not be unequivocally regarded to DCs.

**Table 2:** The summary of studies specifically addressing the role of DCs in MASH.

DC subtype	Diet	Model	Outcomes	Reference
cDC1	MCD	Cd11c.DTR mice	↑ Inflammation ↑ Fibrosis ↑ Hepatocyte apoptosis	(64)
	MCD/HSD	Batf3 KO mice	↑ Inflammation ↑ Macrovesicular steatosis	(65)
	MCCD, CDHFD, WD	-Xcr1.DTR mice -WT mice + anti-XCL1 antibody - Human samples	↓ Inflammation ↓ Hepatocellular damage ↑ DC subsets	(66)
		Human samples (end-stage liver disease)	↓ CD141+ population ↑ IFN-λ production	(56)
Mo-DCs	MCD	WT mice received NaHS	↓ Hepatocellular damage ↓ Apoptosis	(67)

↑, Increase; ↓, Decrease.

A recent study by Deczkowska et al. demonstrated the increased frequency of CD103^+^ XCR1^+^ cDC1 and CD11b^+^ cDC2s in the liver and showed elevated proliferation of DC-committed progenitors in the bone marrow, leading to higher frequencies of circulating pre-DCs in the bloodstream and in the liver (66). Importantly, blocking the infiltration of hepatic cDC1 by using XCL1 antibody in mice fed with CDHFD resulted in a moderate decrease in NASH pathology as compared to the isotype control-treated group (66). These changes included reduction in ALT and NAS score and reduced the presence of accumulated CD8 T cells with the activated/effector memory phenotype (66). Additionally, using a mouse model of XCR1-DTR mice, the loss of cDC1s attenuated immune rearrangements and MASH pathology in the liver (66). Notably, there was increased DC migration and priming activity in draining hepatic lymph node, which suggests superior adaptive T-cell activation that could feed the liver with effector/memory T cells that can become autoaggressive in the metabolically altered milieu. While the study is contradictory to the previous two DC studies, it still could not explain how DCs actually act in MASH. The XCR1-DTR model and DC ablation could simply affect draining LN priming or the lamina propria environment where priming of adaptive immune cells that home towards the liver is generated.

Besides cDCs, Mo-DCs are also increased in MASH liver, and their contribution is even less understood than cDC1s. Specific Mo-DCs expressing CX3CR1 seem to sustain hepatic inflammation by producing TNF. Additionally, ablation of CX3CR1 by hydrogen sulfide (H2S) donor NaHS alleviates parenchymal injury in MASH (67). Notably, the H2S donor and the lack of CX3CR1 affected bone marrow DC differentiation and precursor activity and questioned how this phenomenon could be directly related to the hepatic microenvironment.

## Conclusions and outlook

**Figure 1:**
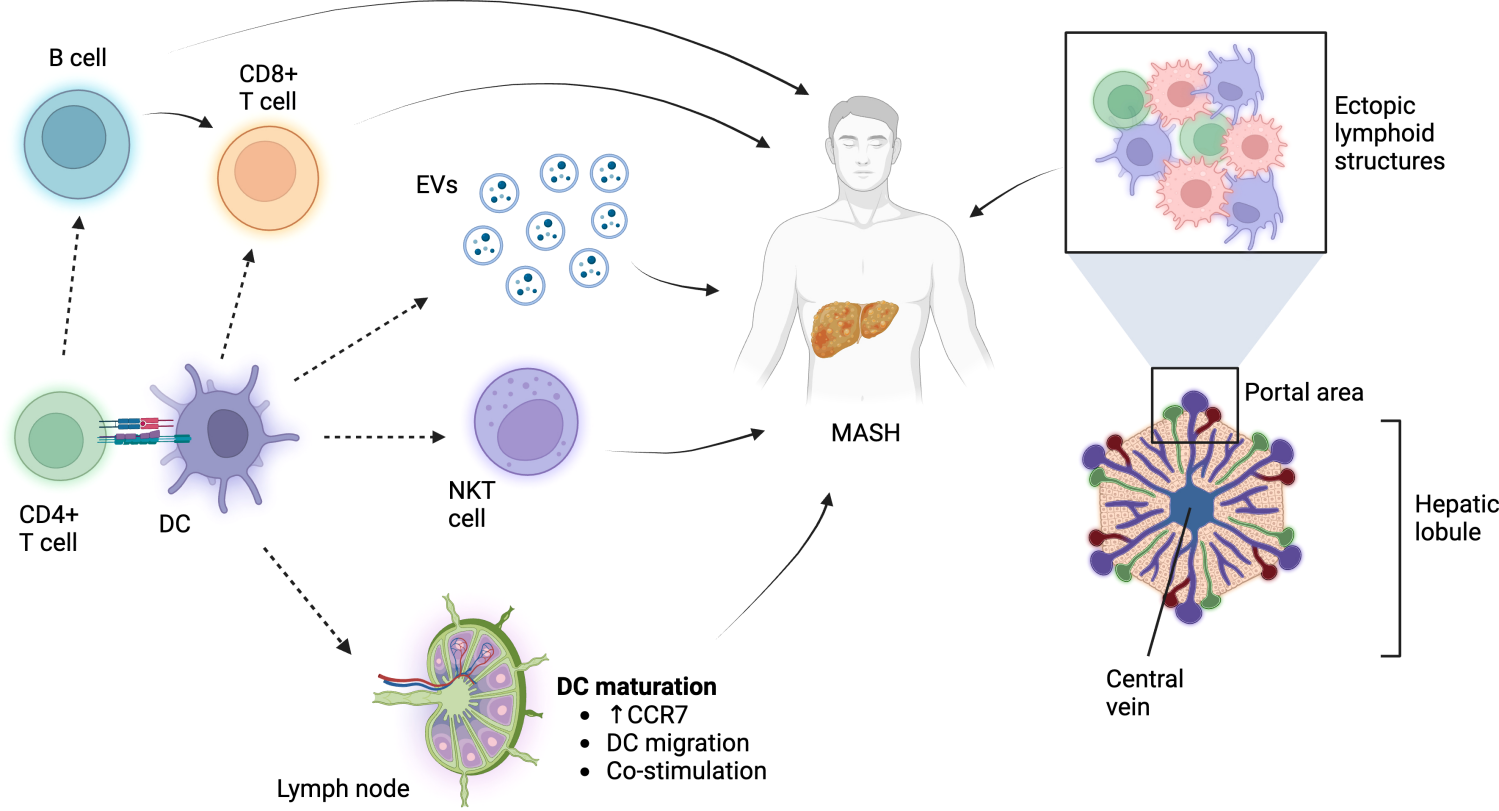
The role of DCs in MASH. DCs influence adaptive and innate immune cells in order to affect MASH pathology. Additionally, they might regulate the pro-anti-inflammatory milieu via soluble molecules, EVs, or immune processes within the ectopic lymphoid structures. Figure was prepared using BioRender.com.

The question “what role do DCs play in MASH?” remains. The answer is most likely a combination of multiple factors (**Figure 1**). Adaptive immune response is key for MASH, and DCs probably contribute (a) as antigen-presenting cells for generating the army of T cells that potentially can home to the liver and (b) in terms of providing CD4 T-cell help for B-cell activation. This would relate to the function of DCs from outside of the liver (in gut and draining LN). Migratory DCs coming from the liver could have significance in bringing oxidative stress-derived antigens and prime naïve T cells. It is also underlined by multiple animal and human studies where the frequency of DCs and their activation state increase (48, 64–66). Increased DC activation has been associated with elevated CCR7 levels, which are vital for DC migration to draining LN (16, 66). Within the liver microenvironment, specifically in the portal region, DCs could regulate the lymphoid aggregates resembling ectopic lymphoid structures as have been described in the lung during flu infection (71). Such structures could feed further T-cell priming without involvement of the lymph node. DCs positioned within or outside of these aggregates, e.g., DCs scattered within the parenchyma, could affect the balance in the inflammatory milieu via secreted molecules or extracellular vesicles. The release of EVs is a major hallmark of matured DCs (72), and secreted EVs have been demonstrated to play a role in liver pathology (73). This could also explain why a relatively low amount of cells (in the context of the total cellularity of the liver) could impede such substantial role on inflammation and disease outcome. Furthermore, DCs could influence innate cells such as NKT cells to affect MASH and liver pathology. It is also important to mention that we do not greatly understand the pre-DC phenotype observed in MASH in the liver. This could gain relevance in the light of other studies demonstrating immensely altered bone marrow hematopoiesis in obesity and during Western diet treatment (74).

Overall, the role of DCs in MASH is currently under investigation, and recent studies have highlighted the potential disease-promoting role of liver DCs in the progression of MASH while other experimental systems suggest their protective role. Further experiments are needed to clarify the exact and likely complex role of DCs in MASH. It will be necessary to use novel technologies such as spatial genomics, proteomics, and metabolomics to better understand the cell-specific effects and niche-specific reprogramming that can influence DCs and liver pathology. Clarifying the role of DCs in MASH holds promise for uncovering novel therapeutic targets and developing more effective treatment strategies for this complex and increasingly prevalent liver condition.

## Author contributions

AP: Writing – original draft, Writing – review & editing, Visualization. VL-K: Writing – original draft, Writing – review & editing, Conceptualization.
